# An average-case sublinear forward algorithm for the haploid Li and Stephens model

**DOI:** 10.1186/s13015-019-0144-9

**Published:** 2019-04-02

**Authors:** Yohei M. Rosen, Benedict J. Paten

**Affiliations:** 1 UCSC Genomics Institute, 1156 High St, Santa Cruz, CA 95064 USA; 2 NYU School of Medicine, 550 First Ave, New York, NY 10016 USA

**Keywords:** Forward algorithm, Haplotype, Complexity, Sublinear algorithms

## Abstract

**Background:**

Hidden Markov models of haplotype inheritance such as the Li and Stephens model allow for computationally tractable probability calculations using the forward algorithm as long as the representative reference panel used in the model is sufficiently small. Specifically, the monoploid Li and Stephens model and its variants are linear in reference panel size unless heuristic approximations are used. However, sequencing projects numbering in the thousands to hundreds of thousands of individuals are underway, and others numbering in the millions are anticipated.

**Results:**

To make the forward algorithm for the haploid Li and Stephens model computationally tractable for these datasets, we have created a numerically exact version of the algorithm with observed average case sublinear runtime with respect to reference panel size *k* when tested against the 1000 Genomes dataset.

**Conclusions:**

We show a forward algorithm which avoids any tradeoff between runtime and model complexity. Our algorithm makes use of two general strategies which might be applicable to improving the time complexity of other future sequence analysis algorithms: sparse dynamic programming matrices and lazy evaluation.

## Background

Probabilistic models of haplotypes describe how variation is shared in a population. One application of these models is to calculate the probability *P*(*o*|*H*), defined as the probability of a haplotype *o* being observed, given the assumption that it is a member of a population represented by a *reference panel* of haplotypes *H*. This computation has been used in estimating recombination rates [1], a problem of interest in genetics and in medicine. It may also be used to detect errors in genotype calls.

Early approaches to haplotype modeling used coalescent [2] models which were accurate but computationally complex, especially when including recombination. Li and Stephens wrote the foundational computationally tractable haplotype model [1] with recombination. Under their model, the probability *P*(*o*|*H*) can be calculated using the forward algorithm for hidden Markov models (HMMs) and posterior sampling of genotype probabilities can be achieved using the forward–backward algorithm. Generalizations of their model have been used for haplotype phasing and genotype imputation [3–7].

### The Li and Stephens model

Consider a *reference panel* *H* of *k* haplotypes sampled from some population. Each haplotype $h_j \in H$ is a sequence $(h_{j,1}, \ldots , h_{j,n})$ of alleles at a contiguous sequence $1, \ldots , n$ of genetic sites. Classically [1], the sites are biallelic, but the model extends to multiallelic sites [8].

Consider an observed sequence of alleles $o = (o_1, \ldots , o_n)$ representing another haplotype. The monoploid Li and Stephens model (LS) [1] specifies a probability that *o* is descended from the population represented by *H*. LS can be written as a hidden Markov model wherein the haplotype *o* is assembled by copying (with possible error) consecutive contiguous subsequences of haplotypes $h_j \in H$.

#### **Definition 1**

(*Li and Stephens HMM*) Define $x_{j,i}$ as the event that the allele $o_i$ at site *i* of the haplotype *o* was copied from the allele $h_{j,i}$ of haplotype $h_j \in H$. Take parameters

1 $$\begin{aligned} \rho ^*_{i-1 \rightarrow i}&\qquad \qquad \text {probability of any recombination between sites } i-1 \text { and } i\end{aligned}$$

2 $$\begin{aligned} \mu _i&\qquad \qquad \text {probability of a mutation from one allele to another at site }i \end{aligned}$$

and from them define the transition and recombination probabilities

3 $$\begin{aligned} p(x_{j,i}|x_{j',i-1})&= {\left\{ \begin{array}{ll} 1 - (k - 1)\rho _i &{} \quad \text {if } j = j'\\ \rho _i &{} \quad \text {if } j \ne j' \end{array}\right. }&\text {where } \rho _i = \frac{\rho ^*_{i-1 \rightarrow i}}{k - 1}\end{aligned}$$

4 $$\begin{aligned} p(o_i|x_{j,i})&= {\left\{ \begin{array}{ll} 1 - (A - 1)\mu _i &{} \quad \text {if } o_i = h_{j,i}\\ \mu _i &{} \quad \text {if } o_i \ne h_{j,i} \end{array}\right. }&\text {where } A = \text {number of alleles} \end{aligned}$$

We will write $\mu _i(j)$ as shorthand for $p(o_i|x_{j,i})$. We will also define the values of the initial probabilities $p(x_{j,1}, o_1 | H) = \frac{\mu _1(j)}{k}$, which can be derived by noting that if all haplotypes have equal probabilities $\frac{1}{k}$ of randomly being selected, and that this probability is then modified by the appropriate emission probability.

Let *P*(*o*|*H*) be the probability that haplotype *o* was produced from population *H*. The forward algorithm for hidden Markov models allows calculation of this probability in $\mathcal {O}(nk^2)$ time using an $n \times k$ dynamic programming matrix of *forward states*

5 $$\begin{aligned} p_i[j] = P(x_{j,i}, o_1, \ldots , o_i | H) \end{aligned}$$

The probability P(*o*|*H*) will be equal to the sum $\sum _j p_n[j]$ of all entries in the final column of the dynamic programming matrix. In practice, the Li and Stephens forward algorithm is $\mathcal {O}(nk)$ (see "Efficient dynamic programming" section).

#### Li and Stephens like algorithms for large populations

The $\mathcal {O}(nk)$ time complexity of the forward algorithm is intractable for reference panels with large size *k*. The UK Biobank has amassed $k = 500,000$ array samples. Whole genome sequencing projects, with a denser distribution of sites, are catching up. Major sequencing projects with $k = 100,000$ or more samples are nearing completion. Others numbering *k* in the millions have been announced. These large population datasets have significant potential benefits: They are statistically likely to more accurately represent population frequencies and those employing genome sequencing can provide phasing information for rare variants.

In order to handle datasets with size *k* even fractions of these sizes, modern haplotype inference algorithms depend on models which are simpler than the Li and Stephens model or which sample subsets of the data. For example, the common tools Eagle-2, Beagle, HAPI-UR and Shapeit-2 and -3 [3–7] either restrict where recombination can occur, fail to model mutation, model long-range phasing approximately or sample subsets of the reference panel.

Lunter’s “fastLS” algorithm [8] demonstrated that haplotypes models which include all *k* reference panel haplotype could find the Viterbi maximum likelihood path in time sublinear in *k*, using preprocessing to reduce redundant information in the algorithm’s input. However, his techniques do not extend to the forward and forward–backward algorithms.

### Our contributions

We have developed an arithmetically exact forward algorithm whose expected time complexity is a function of the expected allele distribution of the reference panel. This expected time complexity proves to be significantly sublinear in reference panel size. We have also developed a technique for succinctly representing large panels of haplotypes whose size also scales as a sublinear function of the expected allele distribution.

Our forward algorithm contains three optimizations, all of which might be generalized to other bioinformatics algorithms. In "Sparse representation of haplotypes" section, we rewrite the reference panel as a sparse matrix containing the minimum information necessary to directly infer all allele values. In "Efficient dynamic programming" section, we define recurrence relations which are numerically equivalent to the forward algorithm but use minimal arithmetic operations. In "Lazy evaluation of dynamic programming rows", we delay computation of forward states using a lazy evaluation algorithm which benefits from blocks of common sequence composed of runs of major alleles. Our methods apply to other models which share certain redundancy properties with the monoploid Li and Stephens model.

## Sparse representation of haplotypes

The forward algorithm to calculate the probability *P*(*o*|*H*) takes as input a length *n* vector *o* and a $k \times n$ matrix of haplotypes *H*. In general, any algorithm which is sublinear in its input inherently requires some sort of preprocessing to identify and reduce redundancies in the data. However, the algorithm will truly become effectively sublinear if this preprocessing can be amortized over many iterations. In this case, we are able to preprocess *H* into a sparse representation which will on average contain better than $\mathcal {O}(nk)$ data points.

This is the first component of our strategy. We use a variant of column-sparse-row matrix encoding to allow fast traversal of our haplotype matrix *H*. This encoding has the dual benefit of also allowing reversible size compression of our data. We propose that this is one good general data representation on which to build other computational work using very large genotype or haplotype data. Indeed, extrapolating from our single-chromosome results, the 1000 Genomes Phase 3 haplotypes across all chromosomes should simultaneously fit uncompressed in 11 GB of memory.

We will show that we can evaluate the Li and Stephens forward algorithm without needing to uncompress this sparse matrix.

### Sparse column representation of haplotype alleles

Consider a biallelic genetic site *i* with alleles $\{A, B\}$. Consider the vector $h_{1,i},$ $h_{2,i}, \ldots , h_{k,i}$ $\in \{A,B\}^k$ of alleles of haplotypes *j* at site *i*. Label the allele *A*, *B* which occurs more frequently in this vector as the major allele 0, and the one which occurs less frequently as the minor allele 1. We then encode this vector by storing the value *A* or *B* of the major allele 0, and the indices $j_1, j_2, \ldots$ of the haplotypes which take on allele value 1 at this site.

We will write $\phi _i$ for the subvector $h_{j_1,i}, h_{j_2,i}, \ldots$ of alleles of haplotypes consisting of those haplotypes which possess the minor allele 1 at site *i*. We will write $|\phi _i|$ for the multiplicity of the minor allele. We call this vector $\phi _i$ the *information content* of the haplotype cohort *H* at the site *i*.

### Relation to the allele frequency spectrum

Our sparse representation of the haplotype reference panel benefits from the recent finding [9] that the distribution over sites of minor allele frequencies is biased towards low frequencies.^1^

Clearly, the distribution of $|\phi _i|$ is precisely the allele frequency spectrum. More formally,

#### **Lemma 1**

*Let* $\mathbb {E}[\overline{f}](k)$ *be the expected mean minor allele frequency for* *k* *genotypes. Then*

6 $$\begin{aligned} \mathbb {E}\left[ \frac{1}{n}\sum _{i = 1}^{n} \left| \phi _i\right| \right] = \mathbb {E}[\overline{f}](k) \end{aligned}$$

#### **Corollary 1**

*If* $\mathcal {O}(\mathbb {E}[\overline{f}]) < \mathcal {O}(k)$, *then* $\mathcal {O}(\sum _i \left| \phi _i\right| ) < \mathcal {O}(nk)$ *in expected value.*

#### Dynamic reference panels

Adding or rewriting a haplotype is constant time per site per haplotype unless this edit changes which allele is the most frequent. It can be achieved by addition or removal or single entries from the row-sparse-column representation, wherein, since our implementation does not require that the column indices be stored in order, these operations can be made $\mathcal {O}(1)$. This allows our algorithm to extend to uses of the Li and Stephens model where one might want to dynamically edit the reference panel. The exception occurs when $\phi _i = \frac{k}{2}$—here it is not absolutely necessary to keep the formalism that the indices stored actually be the minor allele.

### Implementation

**Fig. 1:**
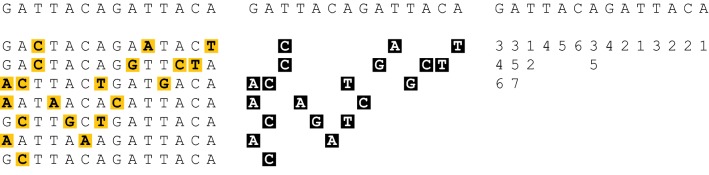
Information content of array of template haplotypes. (i) Reference panel $\{h_1,\ldots ,h_5\}$ with mismatches to haplotype *o* shown in yellow. (ii) Alleles at site *i* of elements of $\phi _i(o_i)$ in black. (iii) Vectors to encode $\phi _i(o_i)$ at each site

For biallelic sites, we store our $\phi _i$’s using a length-*n* vector of length $|\phi _i|$ vectors containing the indices *j* of the haplotypes $h_j \in \phi _i$ and a length-*n* vector listing the major allele at each site (see Fig. 1 panel iii) Random access by key *i* to iterators to the first elements of sets $\phi _i$ is $\mathcal {O}(1)$ and iteration across these $\phi _i$ is linear in the size of $\phi _i$. For multiallelic sites, the data structure uses slightly more space but has the same speed guarantees.

Generating these data structures takes $\mathcal {O}(nk)$ time but is embarrassingly parallel in *n*. Our “*.slls” data structure doubles as a succinct haplotype index which could be distributed instead of a large vcf record (though genotype likelihood compression is not accounted for). A vcf $\rightarrow$ slls conversion tool is found in our github repository.

## Efficient dynamic programming

We begin with the recurrence relation of the classic forward algorithm applied to the Li and Stephens model [1]. To establish our notation, recall that we write $p_i[j] = P(x_{j,i}, o_1, \ldots , o_i | H)$, that we write $\mu _i(j)$ as shorthand for $p(o_i|x_{j,i})$ and that we have initialized ${p_1} [ j ] = p(x_{j,1}, {o_1} | H) = \frac{{{\mu} _1}(j)}{k}$. For $i > 1$, we may then write:

7 $$\begin{aligned} p_i[j]&= \mu _i(j) \left( (1 - k\rho _i) p_{i-1}[j] + \rho _i S_{i-1}\right) \end{aligned}$$

8 $$\begin{aligned} S_{i}&= \sum _{j = 1}^k p_{i}[j] \end{aligned}$$

We will reduce the number of summands in (8) and reduce the number indices *j* for which (7) is evaluated. This will use the *information content* defined in "Sparse column representation of haplotype alleles" section.

### **Lemma 2**

*The summation* (8) *is calculable using strictly fewer than k summands.*

### *Proof*

Suppose first that $\mu _i(j) = \mu _i$ for all *j*. Then

9 $$\begin{aligned} S_{i}&= \sum _{j = 1}^k p_{i}[j] = \mu _i\sum _{j = 1}^k \left( (1 - k\rho _i) p_{i-1}[j] + \rho _i S_{i-1}\right) \end{aligned}$$

10 $$\begin{aligned}&= \mu _i\left( (1 - k\rho _i) S_{i-1} + k\rho _iS_{i-1}\right) = \mu _i S_{i-1} \end{aligned}$$

Now suppose that $\mu _i(j) = 1 - \mu _i$ for some set of *j*. We must then correct for these *j*. This gives us

11 $$\begin{aligned} S_i = \mu _i S_{i-1} + \frac{1 - \mu _i - \mu _i}{1 - \mu _i} \sum _{j \text { where } \mu _i(j) \ne \mu _i} p_{i}[j] \end{aligned}$$

The same argument holds when we reverse the roles of $\mu _i$ and $1 - \mu _i$. Therefore we can choose which calculation to perform based on which has fewer summands. This gives us the following formula:

12 $$\begin{aligned} S_i = \alpha S_{i-1} + \beta \sum _{j \in \phi _i} p_{i}[j] \end{aligned}$$

where

13 $$\begin{aligned} \alpha = \mu _i \quad \beta = \frac{1-2 \mu _i}{1-\mu _i} \quad \text {if } \phi _i \text { have allele a} \end{aligned}$$

14 $$\begin{aligned} \alpha = 1 - \mu _i \quad \beta = \frac{2\mu _i - 1}{\mu _i}\quad\text {if } \phi _i \text { do not have allele a} \end{aligned}$$

$$\square$$

We note another redundancy in our calculations. For the proper choices of $\mu '_i, \mu ''_i$ among $\mu _i, 1 - \mu _i$, the recurrence relations (7) are linear maps $\mathbb {R} \rightarrow \mathbb {R}$

15 $$\begin{aligned}&f_i : x \longmapsto \mu^\prime_i(1 - k\rho )x + \mu^\prime_i\rho S_{i-1} \end{aligned}$$

16 $$\begin{aligned}&F_i : x \longmapsto \mu^{\prime\prime}_i(1 - k\rho )x + \mu^{\prime\prime}_i\rho S_{i-1} \end{aligned}$$

of which there are precisely two unique maps, $f_i$ corresponding to the recurrence relations for those $x_j$ such that $j \in \phi _i$, and $F_i$ to those such that $j \notin \phi _i$.

### **Lemma 3**

*If* $j \notin \phi _i$ *and* $j \notin \phi _{i-1}$, *then* $S_i$ *can be calculated without knowing* $p_{i-1}[j]$ *and* $p_i[j]$. *If* $j \notin \phi _{i-1}$ *and* $j' \ne j$, *then* $p_i[j']$ *can be calculated without knowing* $p_{i-1}[j]$.

### *Proof*

**Fig. 2:**
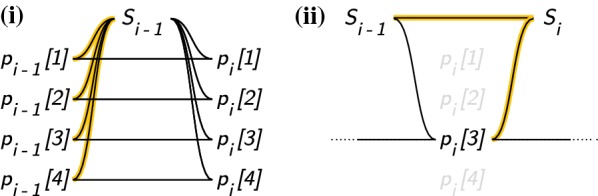
Work done to calculate the sum of haplotype probabilities at a site for the conventional and our sublinear forward algorithm. Using the example that at site *i*, $\phi _i(o_i) = \{h_3\}$, we illustrate the number of arithmetic operations used in (**i**) the conventional $\mathcal {O}(nk)$ Li and Stephens HMM recurrence relations. **ii** Our procedure specified in Eq. (12). Black lines correspond to arithmetic operations; operations which cannot be parallelized over *j* are colored yellow

Equation (12) lets us calculate $S_{i-1}$ without knowing any $p_{i-1}[j]$ for any $j \notin \phi _{i-1}$. From $S_{i-1}$ we also have $f_i$ and $F_i$. Therefore, we can calculate $p_i[j'] = f_i(p_{i-1}[j'])\,or\,F_i(p_{i-1}[j'])$ without knowing $p_{i-1}[j]$ provided that $j' \ne j$. This then shows us that we can calculate $p_i[j']$ for all $j' \in \phi _i$ without knowing any *j* such that $j \notin \phi _i$ and $j \notin \phi _{i-1}$. Finally, the first statement follows from another application of (12) (Fig. 2). $\square$

### **Corollary 2**

*The recurrences* (8) *and the minimum set of recurrences* (7) *needed to compute* (8) *can be evaluated in* $\mathcal {O}(|\phi _i|)$ *time, assuming that* $p_{i-1}[j]$ *have been computed* $\forall j \in \phi _i$.

We address the assumption on prior calculation of the necessary $p_{i-1}[j]$’s in "Lazy evaluation of dynamic programming rows" section.

### Time complexity

Recall that we defined $\mathbb {E}[\overline{f}](k)$ as the expected mean minor allele frequency in a sample of size *k*. Suppose that it is comparatively trivial to calculate the missing $p_{i-1}[j]$ values. Then by Corollary 2 the procedure in Eq. (12) has expected time complexity $\mathcal {O}\left( \sum _i \left| \phi _i\right| \right) = \mathcal {O}\left( n\mathbb {E}[\overline{f}](k)\right)$.

## Lazy evaluation of dynamic programming rows

Corollary 2 was conditioned on the assumption that specific forward probabilities had already been evaluated. We will describe a second algorithm which performs this task efficiently by avoiding performing any arithmetic which will prove unnecessary at future steps.^2^

### Equivalence classes of longest major allele suffixes

#### **Lemma 4**

*Suppose that* $h_j \notin \phi _{\ell } \;\cup \; \phi _{\ell + 1} \;\cup \; \ldots \;\cup \; \phi _{i - 1}$. *Then the dynamic programming* *matrix entries* $p_\ell [j],\; p_{\ell + 1}[j],\; \ldots ,\; p_{i-1}[j]$ *need not be calculated in order to calculate* $S_\ell ,\; S_{\ell + 1},\; \ldots ,\; S_{i-1}$.

#### *Proof*

By repeated application of Lemma (3). $\square$

#### **Corollary 3**

*Under the same assumption on* *j*, $p_\ell [j],\; p_{\ell + 1}[j],\; \ldots ,\; p_{i-1}[j]$ *need not be calculated in order to calculate* $F_{\ell + 1},\; \ldots ,\; F_{i}$. *This is easily seen by definition of* $F_i$.

#### **Lemma 5**

*Suppose that* $p_{\ell - 1}[j]$ *is known, and* $x_j \notin \phi _{\ell } \;\cup \; \phi _{\ell + 1} \;\cup \; \ldots \;\cup \; \phi _{i - 1}$. *Then* $p_{i-1}[j]$ *can be calculated in the time which it takes to* *calculate* $F_{i-1} \circ \ldots \circ F_{\ell }$.

#### *Proof*

$$p_{i-1}[j] = F_{i-1}\circ \ldots \circ F_{\ell }(p_{\ell -1}[j])$$ $$\square$$

It is immediately clear that calculating the $p_i[j]$ lends well to lazy evaluation. Specifically, the $x_j \notin \phi _{i}$ are data which need not be evaluated yet at step *i*. Therefore, if we can aggregate the work of calculating these data at a later iteration of the algorithm, and only if needed then, we can potentially save a considerable amount of computation.

#### **Definition 2**

(*Longest major allele suffix classes*) Define $E_{\ell \rightarrow i - 1} = \phi _{\ell - 1} \cap \left[ \bigcup _{\iota = \ell }^{i - 1} \phi _\iota \right] ^c$ That is, let $E_{\ell \rightarrow i - 1}$ be the class of all haplotypes whose sequence up to site $i - 1$ shares the suffix from $\ell$ to $i - 1$ inclusive consisting only of major alleles, but lacks any longer suffix composed only of major alleles.

#### *Remark 1*

$E_{\ell \rightarrow i - 1}$ is the set of all $h_{j}$ where $p_{\ell - 1}[j]$ was needed to calculate $S_{\ell - 1}$ but no $p_{(\cdot )}[j]$ has been needed to calculate any $S_{(\cdot )}$ since.

Note that for each *i*, the equivalence classes $E_{\ell \rightarrow i-1}$ form a disjoint cover of the set of all haplotypes $h_j \in H$.

#### *Remark 2*

$$\forall h_j \in E_{\ell \rightarrow i - 1}$$, $$p_{i - 1}[j] = F_{i-1}\circ \ldots \circ F_{\ell }(p_{\ell - 1}[j])$$

#### **Definition 3**

Write $F_{a \rightarrow b}$ as shorthand for $F_b \circ \ldots \circ F_a$.

### The lazy evaluation algorithm

Our algorithm will aim to:

1. Never evaluate $p_i[j]$ explicitly unless $h_j \in \phi _i$.
2. Amortize the calculations $p_i[j] = f_i \circ F_{i-1} \circ \ldots \circ F_{\ell }(p_{\ell - 1}[j])$ over all $h_j \in E_{\ell \rightarrow i - 1}$.
3. Share the work of calculating subsequences of compositions of maps $F_{i-1} \circ \ldots \circ F_{\ell }$ with other compositions of maps $F_{i'-1} \circ \ldots \circ F_{\ell '}$ where $\ell ' \le \ell$ and $i' \ge i$.

**Fig. 3:**
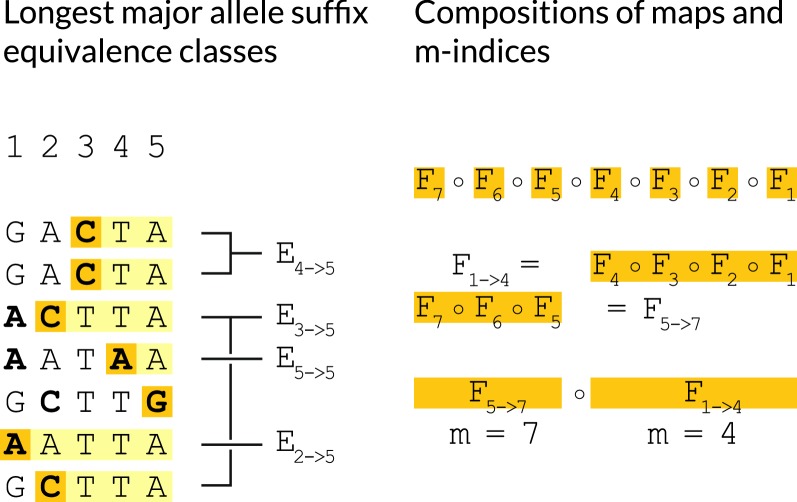
Longest major allele suffix classes, linear map compositions. Illustrations clarifying the meanings of the equivalence classes $E_{\ell \rightarrow i-1}$ (left) and the maps $F_{a\rightarrow b}$. Indices *m* are sites whose indices are *b*’s in stored maps of the form $F_{a\rightarrow b}$

**Fig. 4:**
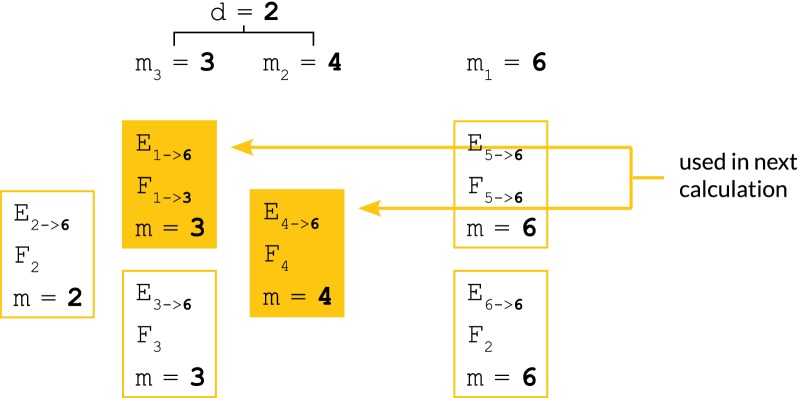
Partial ordering of tuples of (equivalence class, linear map, index) used as state information in our algorithm. The ordering of the tuples $T_\ell = (E_{\ell \rightarrow i -1}, F_{\ell \rightarrow m}, m)$. Calculation of the depth *d* of an update which requires haplotypes contained in the equivalence classes defining the two tuples shown in solid yellow

**Fig. 5:**
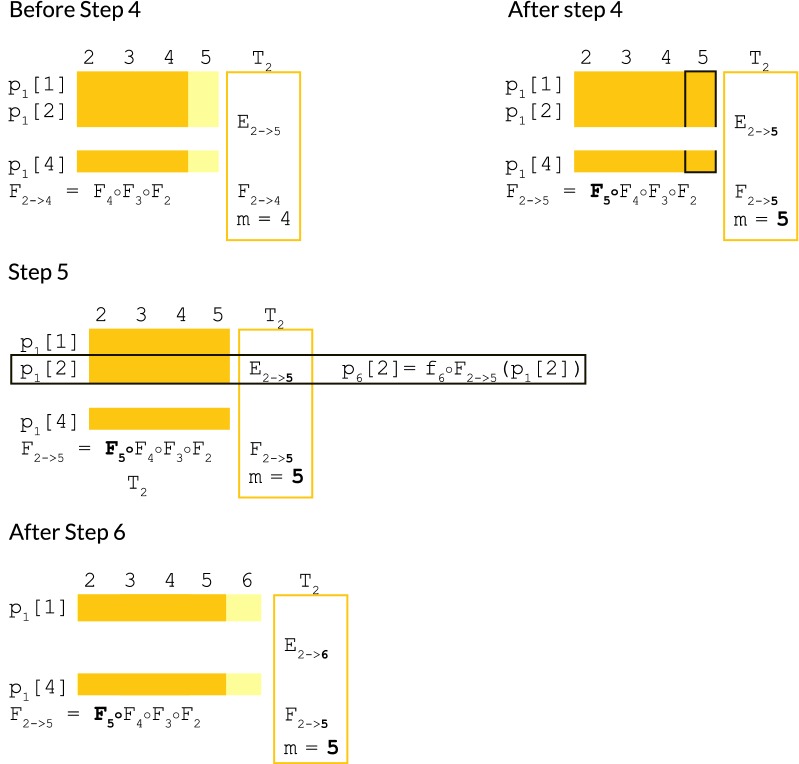
Key steps involved in calculating ${{\varvec{p}}}_{{{\varvec{i}}}}[{{\varvec{j}}}]$ by delayed evaluation. An illustration of the manipulation of the tuple $T_2 = (E_{\ell \rightarrow i-1}, F_{\ell \rightarrow m}, m)$ by the lazy evaluation algorithm, and how it is used to calculate $p_i[j]$ from $p_{\ell -1}[j]$ just-in-time. In this case, we wish to calculate $p_{6}[2]$. This is a member of the equivalence class $E_{2 \rightarrow 5}$, since it hasn’t needed to be calculated since time 1. In step 4 of the algorithm, we therefore must update the whole tuple $T_2$ by post-composing the partially completed prefix $F_{2\rightarrow 4}$ of the map $F_{2 \rightarrow 5}$ which we need using our already-calculated suffix map $F_{5}$. In step 5, we use $F_{2 \rightarrow 5}$ to compute $p_{6}[2] = f_6 \circ F_{2 \rightarrow 5}(p_{1}[j])$. In step 6, we update the tuple $T_2$ to reflect its loss of $h_2$, which is now a member of $E_{6 \rightarrow 6}$

To accomplish these goals, at each iteration *i*, we maintain the following auxiliary data. The meaning of these are clarified by reference to Figs. 3, 4 and 5.

1. The partition of all haplotypes $h_j \in H$ into equivalence classes $E_{\ell \rightarrow i-1}$ according to longest major allele suffix of the truncated haplotype at $i - 1$. See Definition 2 and Fig. 3.
2. The tuples $T_\ell = (E_{\ell \rightarrow i-1}, F_{\ell \rightarrow m}, m)$ of equivalence classes $E_{\ell \rightarrow i-1}$ stored with linear map prefixes $F_{\ell \rightarrow m} =$ $F_{m} \circ \ldots \circ F_\ell$ of the map $F_{\ell \rightarrow i - 1}$ which would be necessary to fully calculate $p_{i}[j]$ for the *j* they contain, and the index *m* of the largest index in this prefix. See Fig. 5.
3. The ordered sequence $m_1> m_2 > \ldots$, in reverse order, of all distinct $1 \le m \le i - 1$ such that *m* is contained in some tuple. See Figs. 3, 5.
4. The maps $F_{min\{\ell \} \rightarrow m_{min}},\;\ldots ,$ $F_{m_2 + 1 \rightarrow m_1},$ $F_{m_1 + 1 \rightarrow i - 1}$ which partition the longest prefix $F_{i-1} \circ \ldots \circ F_{min\{\ell \}}$ into disjoint submaps at the indices *m*. See Fig. 3. These are used to rapidly extend prefixes $F_{\ell \rightarrow m}$ into prefixes $F_{\ell \rightarrow i - 1}$.

Finally, we will need the following ordering on tuples $T_\ell$ to describe our algorithm:

#### **Definition 4**

Impose a partial ordering < on the $T_\ell = (E_{\ell \rightarrow i - 1}, F_{\ell \rightarrow m}, m)$ by $T_\ell < T_{\ell '}$ iff $m < m'$. See Fig. 4.

We are now ready to describe our lazy evaluation algorithm which evaluates $p_i[j] = f_{i} \circ F_{\ell \rightarrow i- 1}(p_{\ell - 1}[j])$ just-in-time while fulfilling the aims listed at the top of this section, by using the auxiliary state data specified above.

The algorithm is simple but requires keeping track of a number of intermediate indices. We suggest referring to the Figs. 3, 4 and 5 as a visual aid. We state it in six steps as follows.

Step 1: Identifying the tuples containing $\phi$—$\mathcal {O}(\phi _i)$ time complexity

Identify the subset $U(\phi )$ of the tuples $T_\ell$ for which there exists some $h_j \in \phi _i$ such that $h_j \in E_{\ell \rightarrow i-1}$.

Step 2: Identifying the preparatory map suffix calculations to be performed—$\mathcal {O}(\phi _i)$ time complexity

Find the maximum depth *d* of any $T_\ell \in U(\phi )$ with respect to the partial ordering above. Equivalently, find the minimum *m* such that $T_\ell = (E_{\ell \rightarrow i - 1}, F_{\ell \rightarrow m}, m) \in U(\phi )$. See Fig. 4.

Step 3: Performing preparatory map suffix calculations—$\mathcal {O}(d)$ time complexity

1. $\mathcal {O}(d)$: Let $m_1, \ldots , m_d$ be the last *d* indices *m* in the reverse ordered list of indices $m_1, m_2, \ldots$. By iteratively composing the maps $F_{m_1 + 1 \rightarrow i -1}, F_{m_2 + 1 \rightarrow m_1}$ which we have already stored, construct the telescoping suffixes $F_{m_1 + 1 \rightarrow i-1},$ $F_{m_2 + 1 \rightarrow i-1}, \ldots ,$ $F_{m_d + 1 \rightarrow i-1}$ needed to update the tuples $(E_{\ell \rightarrow i - 1}, F_{\ell \rightarrow m}, m)$ to $(E_{\ell \rightarrow i - 1}, F_{\ell \rightarrow i - 1}, i - 1)$.
2. $\mathcal {O}(d)$: For each $m_1 \le m_i \le m_d$, choose an arbitrary $(E_{\ell \rightarrow i - 1}, F_{\ell \rightarrow m_i}, m_i)$ and update it to $(E_{\ell \rightarrow i - 1}, F_{\ell \rightarrow i - 1}, i - 1)$.

Step 4: Performing the deferred calculations for the tuples containing $h_j \in \phi _i$—$\mathcal {O}(\phi _i)$ time complexity

If not already done in Step 3.2, for every $T_\ell \in U(\phi )$, extend its map element from $(E_{\ell \rightarrow i - 1}, F_{\ell \rightarrow m}, m)$ to $(E_{\ell \rightarrow i - 1}, F_{\ell \rightarrow i - 1}, i - 1)$ in $\mathcal {O}(1)$ time using the maps calculated in Step 3.1. See Fig. 5.

Step 5: Calculating $p_i[j]$ *just-in-time*—$\mathcal {O}(\phi _i)$ time complexity

*Note:* The calculation of interest is performed here.

Using the maps $F_{\ell \rightarrow i - 1}$ calculated in Step 3.2 or 4, finally evaluate the value $p_i[j] = f_i \circ F_{\ell \rightarrow i -1}(p_{\ell - 1}[j])$. See Fig. 5.

Step 6: Updating our equivalence class/update map prefix tuple auxiliary data structures—$\mathcal {O}(\phi _i + d)$ time complexity

1. Create the new tuple $(E_{i \rightarrow i}, F_{i \rightarrow i} = \text { identity map }, i)$.
2. Remove the $h_j \in \phi _i$ from their equivalence classes $E_{\ell \rightarrow i - 1}$ and place them in the new equivalence class $E_{i \rightarrow i}$. If this empties the equivalence class in question, delete its tuple. *To maintain memory use bounded by number of haplotypes, our implementation uses an object pool to store these tuples*.
3. If an index $m_i$ no longer has any corresponding tuple, delete it, and furthermore replace the stored maps $F_{m_{i-1} + 1 \rightarrow m_i}$ and $F_{m_i + 1} \rightarrow m_{i + 1}$ with a single map $F_{m_{i-1} + 1 \rightarrow m_{i+1}}$. *This step is added to reduce the upper bound on the maximum possible number of compositions of maps which are performed in any given step.*

The following two trivial lemmas allow us to bound *d* by *k* such that the aggregate time complexity of the lazy evaluation algorithm cannot exceed $\mathcal {O}(nk)$. Due to the irregularity of the recursion pattern used by the algorithm, is likely not possible to calculate a closed-form tight bound on $\sum _i d$, however, empirically it is asymptotically dominated by $\sum _i \phi _i$ as shown in the results which follow.

#### **Lemma 6**

*The number of nonempty equivalence classes* $E_{\ell \rightarrow i-1}$ *in existence at any iteration* *i* *of the algorithm is bounded by the number of haplotypes* *k*.

#### *Proof*

Trivial but worth noting. $\square$

#### **Lemma 7**

*The number of unique indices* *m* *in existence at any iteration* *i* *of the algorithm is bounded by the number of nonempty equivalence* *classes* $E_{\ell \rightarrow i-1}$.

## Results

### Implementation

Our algorithm was implemented as a C++ library located at https://github.com/yoheirosen/sublinear-Li-Stephens. Details of the lazy evaluation algorithm will be found there.

We also implemented the linear time forward algorithm for the haploid Li and Stephens model in C++ as to evaluate it on identical footing. Profiling was performed using a single Intel Xeon X7560 core running at 2.3 GHz on a shared memory machine. Our reference panels *H* were the phased haplotypes from the 1000 Genomes [10] phase 3 vcf records for chromosome 22 and subsamples thereof. Haplotypes *o* were randomly generated simulated descendants.

### Minor allele frequency distribution for the 1000 Genomes dataset

**Fig. 6:**
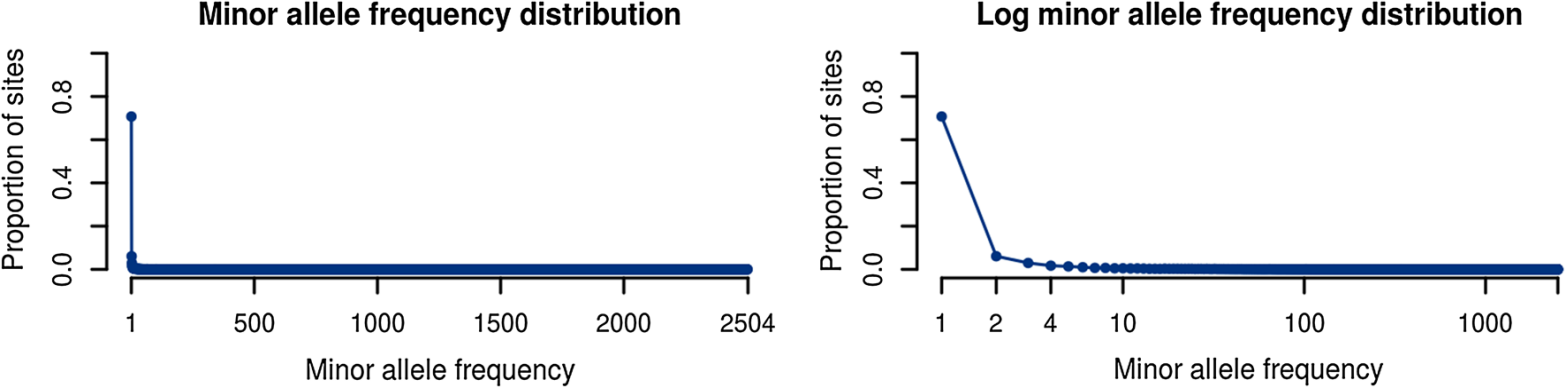
Biallelic site minor allele frequency distribution from 1000 Genomes chromosome 22. Note that the distribution is skewed away from the $\frac{1}{f}$ distribution classically theorized. The data used are the genotypes of the 1000 Genomes Phase 3 VCF, with minor alleles at multiallelic sites combined

We found it informative to determine the allele frequency spectrum for the 1000 Genomes dataset which we will use in our performance analyses. We simulated haplotypes *o* of 1,000,000 bp length on chromosome 22 and recorded the sizes of the sets $\phi _i(o_i)$ for $k = 5008$. These data produced a mean $|\phi _i(o_i)|$ of 59.9, which is 1.2% of the size of *k*. We have plotted the distribution of $|\phi _i(o_i)|$ which we observed from this experiment in (Fig. 6). It is skewed toward low frequencies; the minor allele is unique at 71% of sites, and it is below 1% frequency at 92% of sites.

### Comparison of our algorithm with the linear time forward algorithm

In order to compare the dependence of our algorithm’s runtime on haplotype panel size *k* against that of the standard linear LS forward algorithm, we measured the CPU time per genetic site of both across a range of haplotype panel sizes from 30 to 5008. This analysis was achieved as briefly described above. Haplotype panels spanning the range of sizes from 30 to 5008 haplotypes were subsampled from the 1000 Genomes phase 3 vcf records and loaded into memory in both uncompressed and our column-sparse-row format. Random sequences were sampled using a copying model with mutation and recombination, and the performance of the classical forward algorithm was run back to back with our algorithm for the same random sequence and same subsampled haplotype panel. Each set of runs was performed in triplicate to reduce stochastic error.

**Fig. 7:**
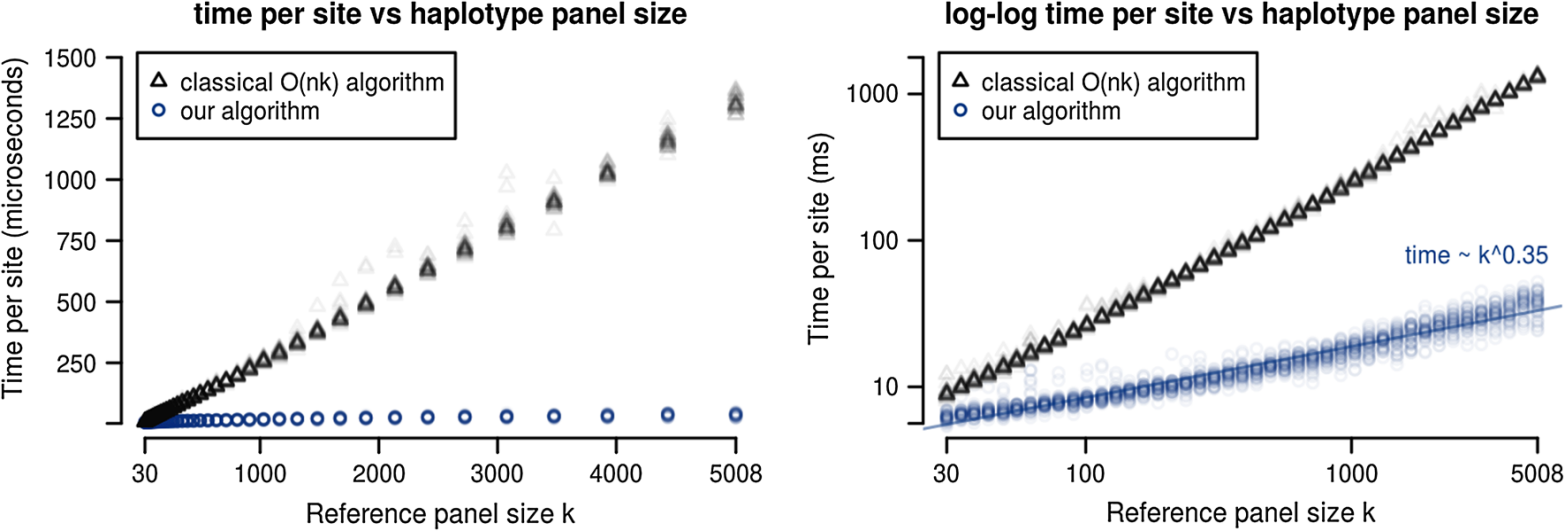
Runtime per site for conventional linear algorithm vs our sparse-lazy algorithm. Runtime per site as a function of haplotype reference panel size k for our algorithm (blue) as compared to the classical linear time algorithm (black). Both were implemented in C++ and benchmarked using datasets preloaded into memory. Forward probabilities are calculated for randomly generated haplotypes simulated by a recombination–mutation process, against random subsets of the 1000 genomes dataset

Figure 7 shows this comparison. Observed time complexity of our algorithm was $\mathcal {O}(k^{0.35})$ as calculated from the slope of the line of best fit to a log–log plot of time per site versus haplotype panel size.

For data points where we used all 1000 Genomes project haplotypes ($k = 5008$), on average, time per site is 37 μs for our algorithm and 1308 μs for the linear LS algorithm. For the forthcoming 100,000 Genomes Project, these numbers can be extrapolated to 251 μs for our algorithm and 260,760 μs for the linear LS algorithm.

#### Lazy evaluation of dynamic programming rows

**Fig. 8:**
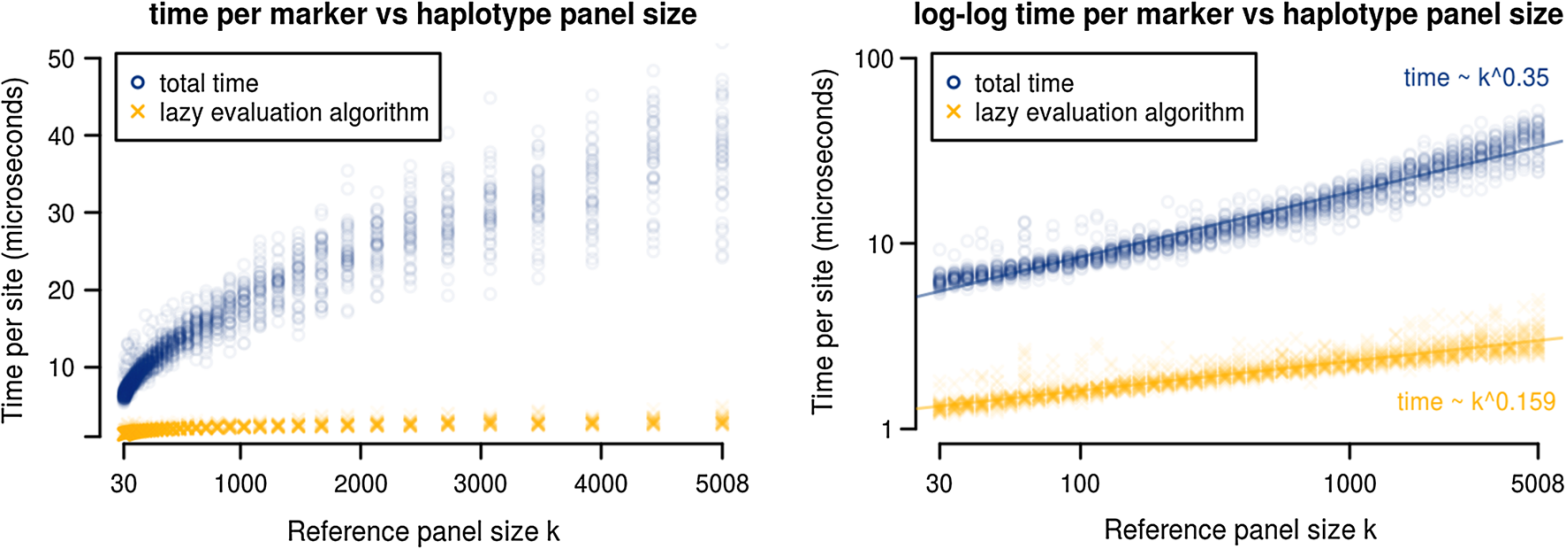
Runtime per site for the overall algorithm and for the recursion-depth dependent portion. Time per site for the lazy evaluation subalgorithm (yellow) vs. the full algorithm (blue). The experimental setup is the same as previously described, with the subalgorithm time determined by internally timing the recursion-depth *d* dependent portions of the lazy evaluation subalgorithm.

We also measured the time which our algorithm spent within the *d*-dependent portion of the lazy evaluation subalgorithm. In the average case, the time complexity of our lazy evaluation subalgorithm does not contribute to the overall algebraic time complexity of the algorithm (Fig. 8, right). The lazy evaluation runtime also contributes minimally to the total actual runtime of our algorithm (Fig. 8, left).

### Sparse haplotype encoding

#### Generating our sparse vectors

We generated the haplotype panel data structures from "Sparse representation of haplotypes" section using the vcf-encoding tool vcf2slls which we provide. We built indices with multiallelic sites, which increases their time and memory profile relative to the results in "Minor allele frequency distribution for the 1000 Genomes dataset" section but allows direct comparison to vcf records. Encoding of chromosome 22 was completed in 38 min on a single CPU core. Use of *M* CPU cores will reduce runtime proportional to *M*.

#### Size of sparse haplotype index

In uncompressed form, our whole genome *.slls index for chromosome 22 of the 1000 genomes dataset was 285 MB in size versus 11 GB for the vcf record using uint16_t’s to encode haplotype ranks. When compressed with gzip, the same index was 67 MB in size versus 205 MB for the vcf record.

In the interest of speed (both for our algorithm and the $\mathcal {O}(nk)$ algorithm) our experiments loaded entire chromosome sparse matrices into memory and stored haplotype indices as uint64_t’s. This requires on the order of 1 GB memory for chromosome 22. For long chromosomes or larger reference panels on low memory machines, the algorithm can operate by streaming sequential chunks of the reference panel.

## Discussions and Conclusion

To the best of our knowledge, ours is the first forward algorithm for any haplotype model to attain sublinear time complexity with respect to reference panel size. Our algorithms could be incorporated into haplotype inference strategies by interfacing with our C++ library. This opens the potential for tools which are tractable on haplotype reference panels at the scale of current 100,000 to 1,000,000+ sample sequencing projects.

### Applications which use individual forward probabilities

Our algorithm attains its runtime specifically for the problem of calculating the single overall probability $P(o|H,\rho ,\mu )$ and does not compute all *nk* forward probabilities. We can prove that if *m* many specific forward probabilities are also required as output, and if the time complexity of our algorithm is $\mathcal {O}(\sum _i\left| \phi _i \right| )$, then the time complexity of the algorithm which also returns the *m* forward probabilities is $\mathcal {O}(\sum _i\left| \phi _i \right| + m)$.

In general, haplotype phasing or genotype imputation tools use stochastic traceback or other similar sampling algorithms. The standard algorithm for stochastic traceback samples states from the full posterior distribution and therefore requires all forward probabilities. The algorithm output and lower bound of its speed is therefore $\mathcal {O}(nk)$. The same is true for many applications of the forward–backward algorithm.

There are two possible approaches which might allow runtime sublinear in *k* for these applications. Using stochastic traceback as an example, first is to devise an $\mathcal {O}(f(m))$ sampling algorithm which uses $m = g(k)$ forward probabilities such that $\mathcal {O}(f \circ g(k)) < \mathcal {O}(k)$. The second is to succinctly represent forward probabilities such that nested sums of the *nk* forward probabilities can be queried from $\mathcal {O}(\phi ) < \mathcal {O}(nk)$ data. This should be possible, perhaps using the positional Burrows–Wheeler transform [11] as in [8], since we have already devised a forward algorithm with this property for a different model in [12].

### Generalizability of algorithm

The optimizations which we have made are not strictly specific to the monoploid Li and Stephens algorithm. Necessary conditions for our reduction in the time complexity of the recurrence relations are

#### Condition 1

*The number of distinct transition probabilities is constant with respect to number of states* *k*.

#### Condition 2

*The number of distinct emission probabilities is constant with respect to number of states* *k*.

Favourable conditions for efficient time complexity of the lazy evaluation algorithm are

#### Condition 1

*The number of unique update maps added per step is constant with respect to number of states* *k*.

#### Condition 2

*The update map extension operation is composition of functions of a class where composition is constant-time with respect to number of states* *k*.

The reduction in time complexity of the recurrence relations depends on the Markov property, however we hypothesize that the delayed evaluation needs only the semi-Markov property.

#### Other haplotype forward algorithms

Our optimizations are of immediate interest for other haplotype copying models. The following related algorithms have been explored without implementation.

##### *Example 1*

(Diploid Li and Stephens) We have yet to implement this model but expect average runtime at least subquadratic in reference panel size *k*. We build on the statement of the model and its optimizations in [13]. We have found the following recurrences which we believe will work when combined with a system of lazy evaluation algorithms:

##### **Lemma 8**

*The diploid Li and Stephens HMM may be expressed using recurrences of the form*

17 $$\begin{aligned} p_{i}[j_1,j_2] = \alpha _p p_{i-1}[j_1,j_2] + \beta _p(S_{i-1}(j_1) + S_{i-1}(j_2)) + \gamma _p S_{i-1} \end{aligned}$$

*which use on the intermediate sums defined as*

18 $$\begin{aligned} S_{i}&:= \alpha _cS_{i-1} + \beta _c\sum _{j\in \phi _i}S_{i-1}(j) + \gamma _c \sum _{(j_1, j_2) \in \phi _i^2} p_{i-1}[j_1,j_2]&\mathcal {O}(|\phi _i|^2) \end{aligned}$$

19 $$\begin{aligned} S_i(j)&:= \alpha _cS_{i-1} + \beta _cS_{i-1}(j) + \gamma _c\sum _{j_2 \in \phi _i}p_{i-1}[j, j_2]&\text {for } \mathcal {O}(k|\phi _i|) \text { many } j \end{aligned}$$

*where* $\alpha _{(\cdot )}, \beta _{(\cdot )}, \gamma _{(\cdot )}$ *depend only on the diploid genotype* $o_i$.

Implementing and verifying the runtime of this extension of our algorithm will be among our next steps.

##### *Example 2*

(Multipopulation Li and Stephens) [14] We maintain separate sparse haplotype panel representations $\phi ^A_i(o_i)$ and $\phi ^B_i(o_i)$ and separate lazy evaluation mechanisms for the two populations *A* and *B*. Expected runtime guarantees are similar.

This model, and versions for $> 2$ populations, will be important in large sequencing cohorts (such as NHLBI TOPMed) where assuming a single related population is unrealistic.

##### *Example 3*

(More detailed mutation model) It may also be desirable to model distinct mutation probabilities for different pairs of alleles at multiallelic sites. Runtime is worse than the biallelic model but remains average case sublinear.

##### *Example 4*

(Sequence graph Li and Stephens analogue) In [12] we described a hidden Markov model for a haplotype-copying with recombination but not mutation in the context of sequence graphs. Assuming we can decompose our graph into nested sites then we can achieve a fast forward algorithm with mutation. An analogue of our row-sparse-column matrix compression for sequence graphs is being actively developed within our research group.

While a haplotype HMM forward algorithm alone might have niche applications in bioinformatics, we expect that our techniques are generalizable to speeding up other forward algorithm-type sequence analysis algorithms.

## Footnotes

1 We observe similar results in our own analyses in "Minor allele frequency distribution for the 1000 Genomes dataset" section.

2 This approach is known as *lazy evaluation*.
